# Cardiovascular autonomic and peripheral sensory neuropathy in women with obesity

**DOI:** 10.3389/fendo.2024.1386147

**Published:** 2024-07-16

**Authors:** Nóra Keller, János Zádori, Balázs Lippai, Dalma Szöllősi, Virág Márton, Károly Wellinger, Szilvia Lada, Mónika Szűcs, Adrienn Menyhárt, Péter Kempler, István Baczkó, Tamás Várkonyi, Csaba Lengyel, Anna Vágvölgyi

**Affiliations:** ^1^ Central Pharmacy, Albert Szent-Györgyi Medical Centre, University of Szeged, Szeged, Hungary; ^2^ Institute of Reproductive Medicine, Albert Szent-Györgyi Medical Centre, University of Szeged, Szeged, Hungary; ^3^ Department of Medicine, Albert Szent-Györgyi Medical School, University of Szeged, Szeged, Hungary; ^4^ Directorate of Nursing Management and Professional Education, Albert Szent-Györgyi Medical Centre, University of Szeged, Szeged, Hungary; ^5^ Department of Medical Physics and Informatics, Albert Szent-Györgyi Medical School, University of Szeged, Szeged, Hungary; ^6^ Department of Internal Medicine and Oncology, Semmelweis University, Budapest, Hungary; ^7^ Department of Pharmacology and Pharmacotherapy, Centre of Excellence for Interdisciplinary Research, Development and Innovation, Albert Szent-Györgyi Medical School, University of Szeged, Szeged, Hungary

**Keywords:** obesity, BMI, cardiovascular autonomic neuropathy, peripheral sensory neuropathy, body composition

## Abstract

**Introduction:**

A higher incidence of neural dysfunction in people with obesity has been described. We determined the prevalence of neuropathic lesions in obese women and evaluated their potential association with anthropometric and laboratory parameters.

**Patients and methods:**

In our cross-sectional study, we enrolled female patients with obesity and without diabetes before obesity treatment. Voluntary female subjects were controls with a normal body mass index (BMI). Autonomic function was assessed by Ewing’s cardiovascular reflex tests, while comprehensive peripheral neuropathic assessments were conducted utilizing the Neurometer®, Tiptherm®, Monofilament®, and Rydel-Seiffer tuning fork tests. Sudomotor function was assessed by the Neuropad®-test. Body composition was examined using the InBody 770.

**Results:**

71 patients (mean ± SD; age: 36.1 ± 8.3 years; BMI: 40.2 ± 8.5 kg/m^2^) and 36 controls (age: 36.4 ± 13.3 years; BMI: 21.6 ± 2.1 kg/m^2^) were enrolled. Patients had significantly higher systolic (patients vs. controls; 137.5 ± 16.9 vs. 114.6 ± 14.8 mmHg, p<0.001) and diastolic (83.0 ± 11.7 vs.69.8 ± 11.2 mmHg, p<0.001) blood pressure compared to controls. Among autonomic tests, only the heart rate response to Valsalva maneuver (Valsalva-ratio) revealed significant impairment in patients (1.4 ± 0.2 vs. 1.7 ± 0.4, p<0.001). Neurometer® at the median nerve revealed increased current perception threshold (CPT) values at all stimulating frequencies in patients (CPT at 2000 Hz: 204.6 ± 70.9 vs. 168.1 ± 66.9, p=0.013; 250 Hz: 84.4 ± 38.9 vs. 56.5 ± 34.8, p<0.001; CPT at 5 Hz: 58.5 ± 31.2 vs 36.9 ± 29.1, p<0.001). The Rydel-Seiffer tuning fork test has revealed a significant impairment of vibrational sensing on the lower limb in patients (right hallux: 6.8 ± 0.9 vs. 7.4 ± 0.8, p=0.030; left hallux: 6.9 ± 0.8 vs. 7.3 ± 0.9, p=0.029). The Neuropad® testing showed a significant impairment of sudomotor function in women with obesity. A negative correlation was found in patients between BMI and the 25-hydroxy-D3/D2-vitamin levels (r=-0.41, p=0.00126) and a positive correlation between the BMI and resting systolic blood pressure (r=0.26, p=0.0325).

**Conclusion:**

Peripheral sensory neuronal and sudomotor function impairments were detected in female patients with obesity compared to the controls with normal BMI. Cardiovascular autonomic dysfunction was also revealed by the Valsalva-ratio in these patients, suggesting the presence of parasympathetic dysfunction. The negative correlation between BMI and the 25-hydroxy-D3/D2-vitamin highlights the potential deficiency of vitamin D in the population affected by obesity.

## Introduction

Obesity alone has emerged as the second most important metabolic risk factor for neuropathy, following diabetes (1). Obesity and dyslipidemia appear to trigger neuropathy even in the absence of overt diabetes mellitus (2, 3). According to the results of an observational study of bariatric surgery candidate women with severe (grade II and III) obesity and without diabetes, the prevalence of polyneuropathy was 11.6%. Neuronal dysfunction has been described to be associated with systemic arterial hypertension, and in multivariate analysis independently associated with age and the postmenopausal status (4). In a recent cross-sectional, observational study (5) in obese and normoglycemic individuals with a body mass index (BMI) greater than 35 kg/m^2^, a high prevalence of neuropathy was found compared to lean controls. Additionally, waist circumference, but not general obesity, was significantly associated with neuropathy. Previous studies in the United States, Denmark, the Netherlands, and Germany have shown an independent relationship between waist circumference and neuropathy, other anthropometric measures were not assessed (6–10). In a cross-sectional, observational study, the prevalence of polyneuropathy was high in obese individuals, even among those with normoglycemia. Diabetes, prediabetes, and obesity are the likely metabolic drivers of neuropathy (6). Several studies reported an important role of cardiovascular risk factors, such as systolic blood pressure (BP), triglyceride levels, BMI and smoking, in the development of cardiac autonomic neuropathy (CAN) (11–13). A study that investigated the association between extensive anthropometric measurements and neuropathy found that female sex was also significantly associated with neuropathy (5).

The goals of our study were to assess neural function in a non-diabetic female population with obesity, and to provide additional help for risk reduction strategies.

## Subjects and methods

### Study subjects

In our cross-sectional, observational study we included 71 female patients with obesity (mean ± SD; age: 36.1 ± 8.34 years; BMI: 40.2 ± 8.47 kg/m^2^) before undergoing lifestyle changes with or without medical treatment. Additionally, a total of 36 age-matched female volunteers with a normal BMI (age: 36.4 ± 13.25 years; BMI: 21.6 ± 2.13 kg/m^2^) were enrolled as controls. Patients were presented at the Endocrinology and Diabetology Outpatient Clinic of the Department of Medicine, Albert Szent-Györgyi Medical School, University of Szeged, Szeged, Hungary. The measurements were performed from March 2021 to May 2023. The exclusion criteria included cases of previously known or freshly diagnosed diabetes, chronic heart failure, any upcoming planned invasive cardiovascular intervention (percutaneous transluminal coronary angioplasty, coronary artery bypass, valve repair or replacement), uncontrolled hypertension (blood pressure > 160/100 mmHg), chronic renal failure, oncological diseases, exposure to chemical agents, serious cognitive dysfunction and lack of cooperation. All of the participants were of Caucasian origin. The relevant clinical data of the subjects are shown in **Table 1**.

**Table 1:** Relevant clinical data in the two study groups.

	Patients with obesity (n=71)	Controls (n=36)	P-value
Clinical Data			
**Age (year)**	36.1 ± 8.34	36.4 ± 13.25	0.872
**Body Mass Index (kg/m^2^)**	40.2 ± 8.47	21.6 ± 2.13	***<0.001***
**Waist-hip-ratio**	0.85 ± 0.12	0.74 ± 0.05	***0.017***
**Systolic BP (mmHg)**	137.5 ± 16.87	114.6 ± 14.81	***<0.001***
**Diastolic BP (mmHg)**	83.0 ± 11.71	69.8 ± 11.17	***<0.001***
**Smoking history (%)**	17 (24)	6 (17)	0.387
**Alcohol consumption (%)**	10 (14)	10 (28)	0.086
**Impaired glucose tolerance (%)**	13 (18)	0 (0)	***0.006***
**Hypertension (%)**	23 (32)	0 (0)	***<0.001***
**Polycystic ovarian syndrome (%)**	36 (51)	2 (6)	***<0.001***
**Hirsutism (%)**	30 (42)	0 (0)	***<0.001***
**Hypothyroidism (%)**	17 (24)	0 (0)	***<0.001***
**Hypercholesterolemia (%)**	2 (3)	2 (6)	0.498
Medication			
**Metformin (%)**	40 (56)	1 (3)	***<0.001***
**β-blocker (%)**	8 (11)	0 (0)	***0.036***
**ACE inhibitor or ARB (%)**	9 (13)	0 (0)	0.097
**Ca-antagonist (%)**	4 (6)	0 (0)	0.147
**Imidazolidine receptor agonist (%)**	3 (4)	0 (0)	0.211
**Alpha-2 adrenergic receptor agonist**	7 (10)	0 (0)	0.051
**Statin (%)**	2 (3)	2 (6)	0.480
**Diuretics (%)**	5 (7)	0 (0)	0.103

BP, blood pressure; ACE, angiotensin-converting enzyme; ARB, angiotensin-receptor blocker; Ca, calcium; ASA, acetylsalicylic acid; PAI, platelet aggregation inhibitor.P-values below 0.05 level are marked bold and italic.

In the above mentioned time period, a total of 71 female patients and only 11 male patients were presented at our clinic due to obesity. Considering the significant difference in gender distribution, to avoid potential bias, the data of the male patients are not shown in this manuscript and our study focuses on female patients only.

### Ewing’s five standard cardiovascular reflex tests, autonomic score

Autonomic function was characterized with Ewing’s five standard cardiovascular reflex tests (14) in the study. The Ewing-tests are the gold standards for diagnosing autonomic dysfunction; they provide non-invasive, clinically relevant, standardized and reproducible data of autonomic functions. Reflex tests were performed by measuring the blood pressure and obtaining continuous 6-lead ECG signals. The signals were digitized with a multichannel data acquisition system (Cardiosys-A01 software, MDE Heidelberg GMBH, Heidelberg, Germany), the sampling rate was 2 kHz and the data were stored for later analysis.

Three of these tests record heart rate changes when performing specific activities, while the rest measures changes in blood pressure. Heart rate tests reflect changes in parasympathetic function, while those based on blood pressure responses primarily characterize sympathetic function disturbances (15). Heart rate changes were measured during deep inhalation and exhalation, in lying and standing positions with 30/15 ratio, and during and following a Valsalva maneuver (16). Systolic blood pressure changes were measured after standing up from a lying position, while diastolic changes were recorded during gripping with the hand for 3 minutes.

CRTs were scored separately: 0 (normal), 1 (borderline), 2 (abnormal). The overall autonomic score was calculated from the sum of each test result to characterize the severity of AN.

#### Heart rate response to deep breathing

Physiologically, the heart rate increases on inhalation and decreases on exhalation. Patients were instructed to take deep breaths at a rate of six breaths per minute (inhale for five seconds and exhale for five seconds). The difference between the measured maximum and minimum heart rates (beats/min) was calculated during six cycles of breathing.

#### Heart rate response to standing up (30/15 ratio)

Normally, the heart rate increases promptly after standing up from a lying position and at about the 15^th^ heartbeat after standing up, it reaches a peak. After that, relative bradycardia presents in healthy individuals with the lowest rate at around the 30^th^ beat. Patients were lying in a supine position at the beginning of the test then they were asked to stand up while the ECG was recorded continuously. The ratio of the longest R-R interval (around beat 30^th^) and the shortest R-R interval (around beat 15^th^) was calculated and recorded as the 30/15 ratio.

#### Heart rate response to the Valsalva maneuver (Valsalva-ratio)

In healthy individuals, the blood pressure decreases and the heart rate increases during Valsalva maneuver. After the maneuver, the blood pressure increases and the heart rate decreases. Subjects were asked to exhale into a specific manometer through a mouthpiece and hold their breath at 40 mmHg for 15 seconds. During that period of time, ECG was continuously recorded. The ratio of the longest R-R interval following the test and the shortest R-R interval during the maneuver was calculated and recorded as the Valsalva-ratio.

#### Systolic blood pressure response to positional change (from lying to standing up)

Normally, in a standing position, the redistribution of blood to the lower limbs is immediately compensated by vasoconstriction in the peripheral vessels. Marked orthostatic hypotension is an important feature of cardiovascular consequences of neuropathy. The test is performed by measuring the blood pressure in a lying position and after standing up. An orthostatic drop in blood pressure is determined with systolic blood pressure measurements at 10 minutes after lying supine and at 1, 5 and 10 minutes after standing up. The difference between the values measured is noted and the largest difference is recorded as the response to standing up.

#### Diastolic blood pressure response during sustained handgrip

Changes in diastolic blood pressure were measured during sustained handgrip. At first, patients were asked to clamp a hand-held dynamometer with their dominant hand exerting full force so that we could determine the maximal grasping force, and then they were instructed to maintain the grasp for 3 minutes at a constant 30% force level. Blood pressure was measured once every minute on the contralateral, relaxed upper limb and the maximally increased diastolic blood pressure was recorded as the response to sustained handgrip.

### Sensory nerve testing

#### Neurometer®

Sensory function of the peripheral nerves was examined with a Neurometer device (NM-01/CPT Neurometer, MDE Heidelberg GmbH, Heidelberg, Germany). The equipment allows non-invasive, simple testing and provides a possibility for the quantitative analysis of sensory nerve function in different types of nerve fibers (17). Transcutaneous, low voltage, sine-wave electrical stimulation was applied and the current perception threshold (CPT) was determined. This study tested the median and the peroneal nerves. The 1 cm diameter surface electrodes were positioned on the distal phalanx of the index finger and that of the hallux. The electrodes were fastened to intact skin surfaces to avoid peripheral sensory disturbance caused by scars and wounds. The amplitude range of the applied stimuli was 0.01 to 9.99 mA. At the beginning of the test, current intensity was gradually increased until the patient indicated sensation. Subsequently, short, 2 to 5-second stimuli were applied at progressively lower intensities until the minimal intensity of consistent sensation was determined. CPT intensities were assessed at three different stimulation frequencies (2000 Hz, 250 Hz, 5 Hz) for both the upper and the lower limbs, assessing large myelinated, small myelinated and small unmyelinated sensory fiber function.

#### 128-Hz Rydel-Seiffer graduated tuning fork

The 128-Hz Rydel-Seiffer graduated tuning fork was used to evaluate the sense of vibration at the distal end of the radius and at the level of the halluces. Results of the tuning fork examination were compared to age-dependent normal values published by Martina et al. in 1998 (18). On a scale of 1-8, the normal range was 7-8, borderline was 6 and abnormal was 1-5, implying an impaired sense of vibration.

#### Semmes-Weinstein monofilament test®

The Semmes-Weinstein Monofilament Test® using a 10 g monofilament is a simple method for the objective screening for protective sensation loss (19). The test was performed under calm and quiet circumstances and the tested individuals were blinded for the place and way of application of the filament. Five regions of the sole were examined in all candidates: hallux, first metatarsus, second metatarsus, heads of the third and the fifth metatarsus. Unaffected sensation in at least 4 regions was considered normal, while 0-3 were abnormal.

#### Tiptherm®

The Tiptherm® (Tip-Therm GmbH, Düsseldorf, FRG) device can be used for the early diagnosis of polyneuropathy of a symmetrical pattern. It is a pen-shaped instrument with flat sides, which tests temperature sensitivity of the skin (20). It contains a 14 mm diameter plastic cylinder and a 14 mm diameter metal cylinder on each end separately. The examiner touches the skin of the patient randomly with one end for 1 second on both hands and feet. The patient has to report which touch was colder (21). In case of normal temperature sensation (<10°C), the individual can differentiate between the two subjective sensations elicited by the flat surfaces of the Tiptherm®, while subjects with impaired temperature sensation cannot distinguish between the two ends.

#### Sudomotor function testing by Neuropad®

Sudomotor dysfunction, which frequently occurs in autonomic neuropathy, was examined with Neuropad® screening tests for all patients and controls (22). The test can detect neuropathy with very high sensitivity (23) on the basis of the fact that nerve fiber impairment in the distal extremities affects not only sensation, but also perspiration. The adhesive pad of the kit contains blue anhydrous cobalt II chloride salt, which reacts and changes to pink when it absorbs water. The tests were performed at room temperature (23°C) following 10 minutes of rest after the patients took off their shoes and socks. The pads were placed on the soles on both sides between the heads of the first and second metatarsi. The color change was read at 10 minutes after adhesion. Total decoloration to pink was considered normal, a mixed pink and blue color was evaluated as pending, while a total blue color was deemed abnormal.

### Questionnaire for the evaluation of neuropathic complaints

Neuropathic complaints were assessed with a questionnaire. Every individual had to make a statement about the presence or absence of burning, pinprick sensations, numbness, tingling, hypoesthesia, hyperesthesia and also about the intensity of these symptoms and frequency of their occurrence.

### Body composition analysis

The body composition analysis was performed by a dietitian with a bioelectrical impedance analysis using a segmental body composition analyzer device (InBody 770 Body, InBodyUSA, Cerritos, CA). The following parameters were determined and documented: skeletal muscle mass (SMM, kg), free fat mass index (calculated by dividing the fat-free mass by the square of the height in meters, FFMI), percent body fat (the total weight of fat in the body divided by the total body weight and then multiplied by 100 to obtain the percentage, PBF, %), whole body phase angle (represents the relationship between resistance (R) and reactance (Xc) of the electrical current passing through the body, WBPA=arctan{Xc/R},°C), bone mineral content (BMC, kg), visceral fat area (represents the amount of visceral fat in the abdominal region of the body, VFA, cm^2^), basal metabolic rate (BMR, Joule), and InBody score (ranging from 0 to 100, with higher scores generally indicating better body composition and overall health, IBS).

### Laboratory data

The laboratory results with a one-month deviation from the appointment date at the obesity clinic were accepted. In case they were available the following parameters were collected and documented: glucose (mmol/L), hemoglobin A1c% (HbA1c; %), insulin (mIU/L), white blood cell count (G/L), red blood cell count (T/L), hemoglobin (g/L), hematocrit (%), mean cell volume of red blood cells (MCV, fL), thrombocyte (G/L), blood urea nitrogen (mmol/L), creatinine (μmol/L), estimated glomerular filtration rate (eGFR; ml/min/1.73 m^2^), uric acid (μmol/L), corrected calcium (mmol/L), phosphate (mmol/L), sodium (mmol/L), potassium (mmol/L), total protein (g/l), albumin (g/l), total cholesterol (mmol/L), triglyceride (mmol/L), HDL-cholesterol (mmol/L), LDL-cholesterol (mmol/L), aspartate aminotransferase (ASAT/GOT; U/l), alanine aminotransferase (ALAT/GPT; U/L), γ-glutamyl transferase (GGT; U/L), total bilirubin (μmol/L), direct/conjugated bilirubin (μmol/L), alkaline phosphatase (U/L), amylase (U/L), lipase (U/L), C-reactive protein (CRP; mg/L), ferritin (ng/mL), serum iron (μmol/L), thyroid-stimulating hormone (TSH; mIU/L), parathormone (pmol/L), 25-hydroxy-D3- and D2-vitamin (25OHD3/D2-vitamin; nmol/L). The following urinary parameters were collected and documented: total protein (mg/dL), albumin (mg/L), creatinine (μmol/L), albumin/creatinine ratio (ACR; mg/mmol), nitrite, pH, protein, glucose, ketone body, urobilinogen, bilirubin, white blood cell, red blood cell. Homeostatic model assessment for insulin resistance (HOMA-IR) was calculated by using the following formula: fasting glucose (mmol/L) X fasting insulin (mIU/L)/22.5.

### Statistical analysis

Data were reported as the mean ± SD; or as frequencies (n) and percentages (%), where appropriate. Pearson’s chi-square test or Fisher’s exact test was used to analyze categorical data, while the independent sample *t* test was used in the case of continuous data. The connections between the continuous or ordinal variables were examined by Pearson’s or Spearman’s correlation analysis. The power analysis for the transition study was performed using G * Power software (Version 3.1.9.7.) for the calculation of the sample size (University of Düsseldorf, Germany). The calculated sample size was 33 and 69 respectively, control and patient groups, working with an effect size d = 0.7 alpha as a Type I error of 0.05, and a power value of 0.9. Statistical tests were performed using statistical software R (R version 3.6.1, https://www.r-project.org/), values of p<0.05 were considered significant.

## Results

### Clinical data of female patients with obesity and control subjects

Relevant clinical data of female patients with obesity (=patients) and control subjects (=controls) are presented in **Table 1**. Age, smoking, alcohol consumption history, and previously known hypercholesterinemia did not differ significantly between the two groups. The patients had significantly higher resting mean systolic (137.5 ± 16.87 vs. 114.6 ± 14.81 mmHg; p<0.001) and diastolic blood pressure (83.0 ± 11.71 vs. 69.8 ± 11.17 mmHg; p<0.001), and suffered significantly more often from hypertension (23 vs. 0, p<0.001), polycystic ovarian syndrome (36 vs. 2; p<0.001), hirsutism (30 vs. 0; p<0.001), and hypothyroidism (17 vs. 0; p<0.001) than the controls. The occurrence of impaired glucose tolerance in patients with obesity was significantly higher (13 vs. 0; p=0.006) in comparison to the controls. Significantly more patients took metformin (40 vs. 1; p<0.001) and β-blocker (8 vs. 0; p=0.036), but there was no significant difference between the two groups with respect to the usage of angiotensin-converting enzyme inhibitors, or angiotensin receptor blockers, Ca^2+^-channel blockers, imidazolidine or alpha-2 adrenergic receptor agonists, statins, and diuretics.

### Laboratory data of female patients with obesity and control subjects

Relevant clinical data of patients and controls are presented in **Table 2**. White blood cell count, hematocrit, thrombocyte, potassium, glucose, uric acid, triglyceride, ALAT/GPT, GGT, alkaline phosphatase, and CRP values were significantly higher in patients with obesity compared to controls with a normal BMI range. The serum phosphate, albumin, creatinine, HDL-cholesterol, amylase, lipase, and 25OHD3/D2-vitamin levels were significantly lower among patients. Other laboratory parameters, such as sodium, adjusted calcium, magnesium, insulin, HOMA-IR, HbA1c, urea nitrogen, eGFR, total protein, total cholesterol, LDL-cholesterol, ASAT/GOT, total bilirubin, ferritin, iron, TSH, parathormone, urine total protein, urine albumin, urine creatinine, ACR, and routine urine test did not show any significant differences. Due to the individualized nature of ambulatory patient care, not all patients and controls had the collected investigated parameters determined.

**Table 2:** Relevant laboratory data in the two study groups.

	Patients with obesity (n=71)	Controls (n=36)	P-value
**White blood cell count (G/L)**	8.3 ± 1.95 (n=65)	6.6 ± 1.34 (n=34)	***<0.001***
**Hemoglobin (g/L)**	134.6 ± 18.25 (n=66)	130.4 ± 10.07 (n=34)	0.220
**Hematocrit (L/L)**	0.41 ± 0.03 (n=66)	0.39 ± 0.03 (n=34)	***<0.001***
**Mean cellular volume (fL)**	85.9 ± 4.15 (n=66)	86.7 ± 3.84 (n=34)	0.364
**Thrombocyte (G/L)**	313.6 ± 62.95 (n=66)	277.67 ± 71.47 (n=33)	*0.012*
**Sodium (mmol/L)**	139.1 ± 2.25 (n=66)	138.6 ± 2.36 (n=34)	0.346
**Potassium (mmol/L)**	4.6 ± 0.39 (n=66)	4.3 ± 0.34 (n=34)	***<0.001***
**Adjusted calcium (mmol/L)**	2.3 ± 0.09 (n=62)	2.3 ± 0.07 (n=31)	0.309
**Magnesium (mmol/L)**	0.9 ± 0.09 (n=61)	0.9 ± 0.09 (n=27)	0.854
**Phosphate (mmol/L)**	1.0 ± 0.17 (n=57)	1.2 ± 0.27 (n=29)	***<0.001***
**Glucose (mmol/L)**	5.1 ± 0.61 (n=68)	4.7 ± 0.62 (n=33)	***<0.001***
**Insulin (mIU/L)**	17.1 ± 18.97 (n=48)	11.2 ± 14.91 (n=8)	0.400
**HOMA-IR**	4.0 ± 4.79 (n=46)	2.5 ± 3.44 (n=8)	0.383
**HbA1c (%)**	5.4 ± 0.39 (n=67)	5.4 ± 0.33 (n=32)	0.786
**Blood urea nitrogen (mmol/L)**	5.2 ± 6.73 (n=65)	4.3 ± 1.92 (n=35)	0.397
**Creatinine (μmol/L)**	65.2 ± 9.09 (n=64)	69.0 ± 9.37 (n=35)	***0.050***
**eGFR (mL/min/1.73m^2^)**	96.9 ± 28.72 (n=62)	90.0 ± 17.21 (n=33)	0.210
**Uric acid (μmol/L)**	308.4 ± 76.75 (n=60)	219.5 ± 40.81 (n=34)	***<0.001***
**Total protein (g/L)**	72.7 ± 5.48 (n=57)	72.9 ± 4.06 (n=29)	0.831
**Albumin (g/L)**	47.13 ± 2.69 (n=62)	48.5 ± 2.57 (n=35)	***0.017***
**Total cholesterol (mmol/L)**	5.0 ± 0.72 (n=64)	4.9 ± 0.96 (n=35)	0.736
**Triglyceride (mmol/L)**	1.5 ± 0.75 (n=64)	1.0 ± 0.43 (n=35)	***<0.001***
**HDL-cholesterol (mmol/L)**	1.3 ± 0.34 (n=63)	1.8 ± 0.44 (n=34)	***<0.001***
**LDL-cholesterol (mmol/L)**	2.9 ± 0.62 (n=59)	2.6 ± 0.84 (n=35)	0.074
**ASAT/GOT (U/L)**	23.4 ± 16.69 (n=63)	20.9 ± 7.70 (n=34)	0.414
**ALAT/GPT (U/L)**	28.3 ± 14.69 (n=63)	19.5 ± 11.5 (n=34)	***0.003***
**Gamma GT (U/L)**	27.9 ± 20.88 (n=63)	13.1 ± 5.73 (n=34)	***<0.001***
**Total bilirubin (μmol/L)**	7.9 ± 3.49 (n=61)	8.8 ± 5.67 (n=33)	0.360
**Alkaline phosphatase (U/L)**	75.2 ± 20.81 (n=62)	59.7 ± 17.20 (n=34)	***<0.001***
**Amylase (U/L)**	46.6 ± 16.85 (n=46)	72.6 ± 24.98 (n=20)	***<0.001***
**Lipase (U/L)**	30.5 ± 8.69 (n=42)	50.4 ± 13.90 (n=8)	***<0.001***
**C-reactive protein (mg/L)**	8.9 ± 8.84 (n=57)	1.2 ± 3.28 (n=31)	***<0.001***
**Ferritin (ng/mL)**	77.9 ± 55.72 (n=50)	42.9 ± 23.04 (n=7)	0.109
**Iron (μmol/L)**	14.2 ± 5.36 (n=60)	17.0 ± 7.49 (n=20)	0.083
**TSH (mIU/L)**	2.2± 1.27 (n=64)	2.3 ± 1.61 (n=21)	0.849
**Parathormone (pmol/L)**	4.5 ± 5.48 (n=60)	3.2 ± 1.79 (n=14)	0.390
**25OHD3/D2-vitamin (nmol/L)**	63.3 ± 23.51 (n=60)	80.9 ± 28.07 (n=17)	***0.011***
**Urine creatinine (μmol/L)**	13521.3 ± 16938.03 (n=32)	10229.8 ± 5572.89 (n=13)	0.499
**Urine total protein (mg/dL)**	8.4 ± 6.29 (n=36)	7.9 ± 4.99 (n=22)	0.732
**Urine albumin (mg/L)**	12.1 ± 19.0) (n=35)	9.5 ± 16.54 (n=18)	0.620
**ACR (mg/mmol)**	2.0 ± 2.95 (n=25)	1.4 ± 1.99 (n=10)	0.607
**Urine pH**	6.0 ± 0.71	6.0 ± 0.59	0.912
**Urine nitrite, negative (n/%)**	42 (100.0%)	25 (100.0%)	0.999
**Urine protein, negative (n/%)**	35 (81.4%)	22 (78.6%)	0.770
**Urine glucose, negative (n/%)**	43 (100.0%)	28 (100.0%)	0.075
**Urine ketone, negative (n/%)**	41 (97.6%)	27 (96.4%)	0.770
**Urine urobilinogen, negative (n/%)**	43 (100.0%)	27 (96.4%)	0.212
**Urine bilirubin, negative (n/%)**	43 (100.0%)	28 (100.0%)	0.075
**Urine white blood cell, negative (n/%)**	35 (81.4%)	21 (75.0%)	0.519
**Urine red blood cell, negative (n/%)**	38 (90.5%)	24 (85.7%)	0.540

The data are presented as mean ± SD. HOMA-IR, homeostasis model assessment of insulin resistance; HbA1c, hemoglobin A1c; eGFR, estimated glomerular filtration rate; ASAT/GOT, aspartate aminotransferase; ALAT/GPT, alanine aminotransferase; GGT, γ-glutamyl transferase; TSH, thyroid-stimulating hormone; 25OHD3/D2-vitamin, 25-hydroxy-D3- and D2-vitamin; ACR, albumin/creatinine ratio; n, number of the negative results; %: percentage of the negative results compared to the tested total sample.P-values below 0.05 level are marked bold and italic.

### Cardiovascular autonomic function tests of female patients with obesity and control subjects

**Figure 1:**
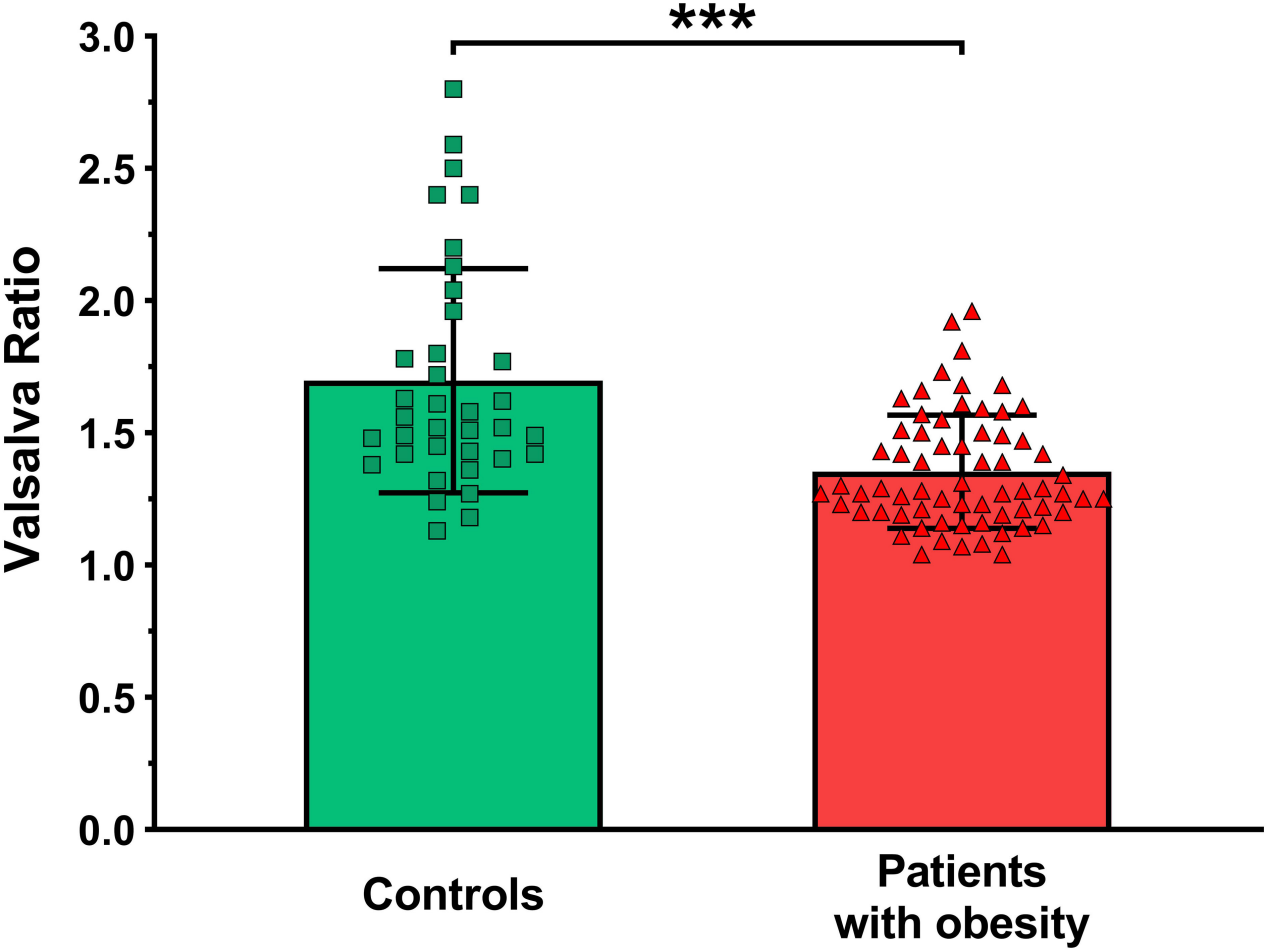
Heart rate responses to Valsalva maneuver (Valsalva-ratio) in the study groups. *** p<0.001.

The results of the cardiovascular autonomic function tests in patients and controls are shown in **Table 3**. A significant impairment in Valsalva-ratio (**Figure 1**) in patients was detected compared to controls (1.4 ± 0.21 vs. 1.7 ± 0.42; p<0.001). No further significant differences in the results of autonomic tests could be detected between the two groups.

**Table 3:** Results of the cardiovascular autonomic function tests in female patients with obesity and controls.

Reflex tests	Patients with obesity (n=69)	Controls (n=36)	P-value
**HRRDB (1/min)**	24.6 ± 8.21	24.0 ± 7.86	0.709
**HRRSU (30/15 ratio)**	1.2 ± 1.22	1.1 ± 0.12	0.596
**VR**	1.4 ± 0.21	1.7 ± 0.42	***<0.001***
**SBPRSU (mm Hg)**	3.7 ± 8.32	3.6 ± 4.39	0.925
**Handgrip (mmHg)**	12.1 ± 9.55	11.2 ± 10.43	0.670
**Autonomic score**	2.25 ± 1.59	1.9 ± 1.30	0.248

HRRDB: the heart rate response to deep breathing; HRRSU (30/15 ratio): the heart rate responses to standing up; VR (Valsalva-ratio): the heart rate responses to Valsalva maneuver; SBPRSU: the systolic blood pressure response to standing up.P-values below 0.05 level are marked bold and italic.

### Peripheral sensory function in female patients with obesity and control subjects

Significant differences were demonstrated in the peripheral sensory function of median nerves in all three tested frequencies with Neurometer®. At the median nerve, testing revealed increased thresholds (**Table 4**) in female patients with obesity versus controls across all tested frequencies (at 2000 Hz: 204.6 ± 70.90 vs. 168.1 ± 66.87 CPT, p=0.013; at 250 Hz: 84.4 ± 38.91 vs. 56.5 ± 34.82 CPT, p<0.001; at 5 Hz: 58.5 ± 31.22 vs. 36.9 ± 29.07 CPT, p<0.001, respectively). At the peroneal nerve, testing also revealed elevated thresholds, but these did not reach the level of significance.

**Table 4:** Peripheral sensory function testing by Neurometer® assessing the threshold of the current sensations at the median and peroneal nerves at three different stimulating frequencies (2 kHz, 250 Hz, 5 Hz).

	Patients with obesity (n=66)	Controls (n=36)	P-value
**NM2000**	204.6 ± 70.90	168.1 ± 66.87	***0.013***
**NM250**	84.4 ± 38.91	56.5 ± 34.82	***<0.001***
**NM5**	58.5 ± 31.22	36.9 ± 29.07	***<0.001***
**NP2000**	341.6 ± 113.76	320.9 ± 114.97	0.385
**NP250**	165.5 ± 89.07	158.8 ± 63.06	0.689
**NP5**	101.5 ± 58.85	98.6 ± 47.88	0.799

NM2000, CPT (current perception threshold) value of the median nerve at stimulating frequency of 2000 Hz; NM250, CPT value of the median nerve at stimulating frequency of 250 H; NM5, CPT value of the median nerve at stimulating frequency of 5 Hz; NP2000, CPT value of the peroneal nerve at stimulating frequency of 2000 Hz; NP250, CPT value of the peroneal nerve at stimulating frequency of 250 Hz; NP5, CPT value of the peroneal nerve at stimulating frequency of 5.P-values below 0.05 level are marked bold and italic.

Peripheral sensory function testing by 128-Hz Rydel-Seiffer graduated tuning fork (**Table 5**) revealed a significant impairment of vibrational sensing on the lower limb in patients. Peripheral sensory function testing by Semmes-Weinstein Monofilament Test® and by Tiptherm® did not reveal significant differences between the two groups.

**Table 5:** Peripheral sensory function testing in the study groups by 128-Hz Rydel-Seiffer graduated tuning fork on the distal end of right (RR) and left (LR) radius and the right (RH) and left (LH) hallux.

	Patients with obesity (n=67)	Controls (n=36)	P-value
**RR**	7.4 ± 0.63	7.5 ± 0.78	0.636
**LR**	7.4 ± 0.69	7.6 ± 0.50	0.087
**RH**	6.8 ± 0.94	7.4 ± 0.80	***0.030***
**LH**	6.9 ± 0.84	7.3 ± 0.94	***0.029***

P-values below 0.05 level are marked bold and italic.

Sudomotor function testing using Neuropad® (**Table 6**) revealed a significantly impaired autonomic function due to decreased perspiration in patients when compared to controls on both the right and left sole.

**Table 6:** Sudomotor function testing with Neuropad® in the study groups.

	Patients with obesity (n=62)	Controls (n=27)	P-value
**Left side**			***0.008***
**Blue**	0 (0.0%)	1 (3.7%)	
**Mixed**	19 (30.6%)	1 (3.7%)	
**Rose**	43 (69.4%)	25 (92.6%)	
**Right side**			***0.035***
**Blue**	1 (1.6%)	1 (3.7%)	
**Mixed**	17 (27.4%)	1 (3.7%)	
**Rose**	44 (71.0%)	25 (92.6%)	

P-values below 0.05 level are marked bold and italic.

### Body composition analysis

All InBody® parameters (**Table 7**) showed significant discrepancies between the two groups in accordance with our preliminary expectations.

**Table 7:** Body composition analysis with InBody® in the study groups.

InBody® parameters	Patients with obesity (n=51)	Controls (n=23)	P-value
**SMM (kg)**	31.9± 4.68	25.0 ± 3.12	***<0.001***
**FFMI (kg/m^2^)**	20.4 ± 2.22	16.0 ± 1.27	***<0.001***
**PBF (%)**	46.5 ± 5.12	23.5 ± 4.73	***<0.001***
**WBPA (°C)**	5.7 ± 0.52	5.3 ± 0.46	***<0.001***
**BMC (kg)**	3.2 ± 0.48	2.7 ± 0.33	***<0.001***
**VFA (cm^2^)**	220.4 ± 50.44	60.9± 16.77	***<0.001***
**IBS**	58.4 ± 8.79	77.2 ± 4.22	***<0.001***

SMM, skeletal muscle mass; FFMI, free fat muscle index; PBF%, percent body fat; WBPA, whole body phase angle; BMC, bone mineral content; VFA, visceral fat area; IBS, in body score.P-values below 0.05 level are marked bold and italic.

### Evaluation of neuropathic symptoms with questionnaire

No statistical difference in neuropathic symptoms was found between the two groups (12{17.6%} vs. 2 {5.6%}, p=0.086).

### Correlations between studied parameters

**Figure 2:**
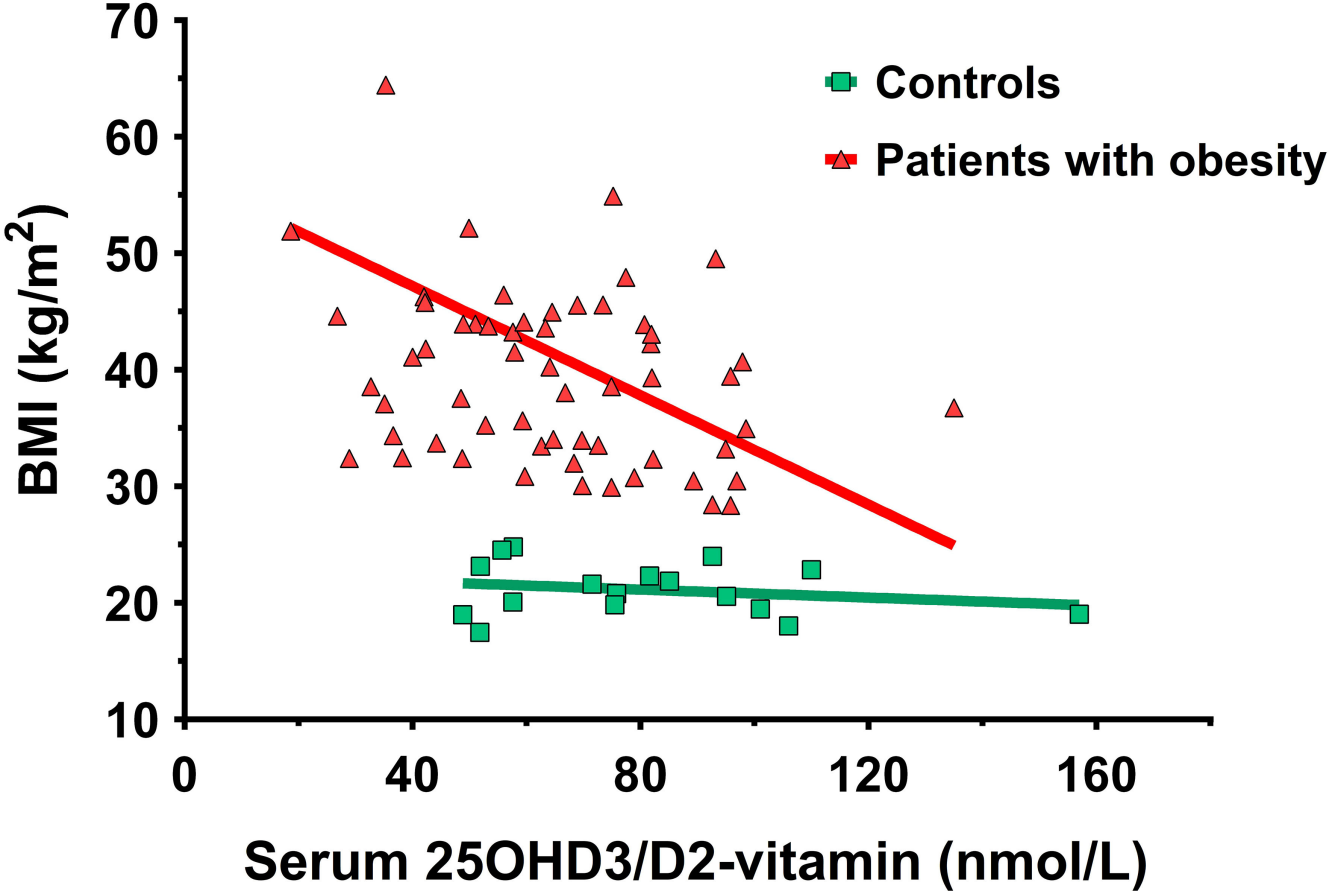
Negative correlation between BMI and the 25-hydroxy-D3- and D2-vitamin level in the female patients with obesity.

Among female patients with obesity, a negative correlation was found between BMI and the levels of 25OHD3/D2-vitamin (r=-0.41, p=0.00126), while a positive correlation was identified between BMI and resting systolic blood pressure (r=0.26, p=0.0325). In patients, the 25OHD3/D2-vitamin level reached a significant, negative correlation with waist circumference (r=-0.26, p=0.0493). A similar correlation could not be confirmed in the case of the controls. In patients with obesity waist (r=0.28, p=0.0214) and hip (r=0.39, p=0.00124) circumference showed a positive correlation with resting systolic blood pressure. This correlation was also observed in controls (r=0.38, p=0.045). In patients, skeletal muscle mass (r=0.41, p=0.00355) and free fat muscle index (r=0.5, p=0.000263), percent body fat % (r=0.29, p=0.0403), and basal metabolic rate (r=0.42, p=0.00265) all correlated positively with resting systolic blood pressure. A similar correlation in controls was found only between percent body fat % and resting systolic blood pressure (r=0.43, p=0.0381), as well as with resting diastolic blood pressure (r=0.46, p=0.0279). Furthermore, in patients with obesity bone mineral content showed a slight positive correlation with the Valsalva-ratio (r=0.27000, p=0.0498); a correlation similar to this was not observed in the controls (**Figure 2**). In patients with obesity, a prominent positive correlation (r=0.56, p=0.00468) was revealed between the CPT value of the median nerve at stimulating frequency of 2000 Hz (NM2000) and the albumin/creatinine ratio (ACR). Additionally, the CPT value of the peroneal nerve at the stimulation frequency of 2000 Hz (NP2000) demonstrated a negative association in patients (r=-0.30000, p=0.0234) with the results of sustained handgrip test. In controls, the CPT value of the median nerve at stimulating frequency of 2000 Hz (NM2000) exhibited a negative correlation with hip circumference (r=-0.43, p=0.0229), while at the stimulation frequency of 5 Hz (NM5), a positive association with the CRP laboratory parameter (r= 0.36, p=0.0461) was revealed.

## Discussion

### Autonomic function in female patients with obesity

Female patients with obesity performed significantly worse Valsalva-tests than controls. It is important to note that the mean Valsalva-ratio in both groups was found within the normal range (≥1.21). However, this identified discrepancy supports an emerging parasympathetic dysfunction in female patients with obesity. This finding can also be explained as an effect of the elevated sympathetic nervous system activity. The revealed slight positive correlation in patients between the bone mineral content and the Valsalva-ratio suggests that better bone health might be associated with a more optimal performance in this cardiovascular reflex test. Several lines of evidence suggest that the autonomic nervous system may play a role in bone remodeling and the maintenance of bone mass, although much of the evidence comes from rodent experiments (24). A few previous studies have shown an association between autonomic dysfunction and reduced bone mineral density (25–28), although the results are contradictory.

Sudomotor dysfunction, which frequently occurs in autonomic neuropathy, was examined with Neuropad® screening tests in the study subjects. The test revealed a decreased perspiration in patients in comparison to controls both on the right and left sole, confirming the deterioration of autonomic function in female patients with obesity. To the best of our knowledge, our finding represents the first documented instance of certified small fiber dysfunction with this methodology in women with obesity. A recent study, without mentioning sex distribution of a small cohort of patients (n=26) with obesity, without diabetes and before bariatric surgery, confirmed that patients with obesity have evidence of small and large fiber neuropathy in comparison to controls (n=20) (29).

### Sympathetic nervous system overdrive in obesity and biometric parameters

It is known that obesity is associated with sympathetic nervous system overdrive, which is considered as a compensatory mechanism for the increase in energy consumption, thus enabling restoration of the energy equilibrium (30, 31). Accordingly, sympathetic nervous system activity was demonstrated to have a correlation with visceral fat accumulation (32). In our patient cohort, waist and hip circumference showed a positive correlation with resting systolic blood pressure. This correlation was similarly observed in the control group. Free fat muscle index and percent body fat also correlated positively with resting systolic blood pressure in obese patients, supporting the correlation between elevated sympathetic nervous system activity and fat content in this population. Excessive activation of the sympathetic nervous system could potentially play a role in metabolic issues, including insulin resistance leading to increased insulin levels, disrupted glucose metabolism, diabetes, and abnormal lipid levels (33, 34).

### Additional factors influencing autonomic function

In our study, the patients with obesity had no diabetes and showed no significant differences in terms of age, smoking, alcohol consumption, and a history of hypercholesterolemia when compared to controls. However, our patients exhibited significantly higher resting mean systolic and diastolic blood pressure levels, and a higher prevalence of hypertension, polycystic ovarian syndrome and hypothyroidism, compared to the control group.

Significantly more patients took metformin and β-blockers, but there were no significant differences between the two groups with respect to the usage of angiotensin-converting enzyme inhibitors, or angiotensin receptor blockers, calcium channel blockers, imidazolidine or α_2_ adrenergic receptor agonists, statins, and diuretics. Beta-adrenergic blockers decrease sympathetic nerve activity, and therefore, they might attenuate the extent of abnormality in the achieved reflex test results in patients. Similarly, with metformin, we can anticipate a comparable masking effect through the optimization of carbohydrate metabolism.

### Peripheral sensory function in female patients with obesity

Significant differences were demonstrated in the peripheral sensory function of median nerves in all three tested frequencies with Neurometer®. Testing the median nerve revealed increased thresholds in female patients with obesity versus controls at all tested frequencies, indicating a deteriorated function in large myelinated, small myelinated and small unmyelinated sensory fibers. Similarly, peroneal nerve testing revealed a tendency of increased thresholds, but these did not reach the level of significance. An increase in case numbers may further confirm the statistically significant difference indicated by the increased CPT values at the peroneal nerve compared to controls with a normal BMI range.

Peripheral sensory function testing by 128-Hz Rydel-Seiffer graduated tuning fork has identified a significant impairment of vibrational sensing of patients on the lower limb. To the best of our knowledge, our study is the first to demonstrate vibrational sensing impairment in women with obesity. The Semmes-Weinstein Monofilament Test® and the Tiptherm® test did not reveal significant differences between the two groups.

### Evaluation of laboratory data of female patients with obesity

As observed in our research, the CRP value was significantly higher in patients with obesity compared to controls with a normal value of BMI. Several studies have shown that elevated CRP is strongly associated with obesity as part of the metabolic syndrome (35–38).

Fröhlich et al. found a definitive association between CRP and BMI in a large population-based sample (35). Choi et al. (36) found a strong association between BMI and CRP in their systematic review, with a stronger association in women compared to men.

In a recent cross-sectional analysis by Cohen et al. (37) white blood cell count, platelet count, erythrocyte sedimentation rate and CRP were assessed in normal, overweight, obese and morbidly obese BMI categories in 7526 men and 3219 women. In subjects with an abnormal BMI range, inflammatory markers were significantly higher compared to those with a normal BMI value, with this difference being more pronounced in women than in men. The theoretically explicable variation in inflammatory markers between genders may be attributed to the different fat disposition (visceral *vs*. subcutaneous) among men and women and the varying influence of sex hormones (36, 37). Body fat may potentially increase CRP concentration in the obese population, as adipose tissue is a major regulator of inflammation, alongside coagulation and fibrinolysis (38). Adipose tissue is an active endocrine organ that produces proinflammatory cytokines such as tumor necrosis factor-α and interleukin-6 (IL-6), which is the main stimulator of the hepatic synthesis of CRP (36, 38, 39). Inflammation can be diminished with drugs and lifestyle modifications as weight loss has been associated with a decrease in CRP and cytokine levels (38). To sum it up, higher white blood cell count and CRP values reflect chronic nonspecific inflammation, suggesting global metabolic disturbances in our patient group.

In our study, hematocrit, white blood cell and thrombocyte counts were all significantly higher in patients with obesity compared to controls with a normal BMI value. In epidemiological studies, although subjects with obesity exhibit relatively higher white blood counts, their absolute leukocyte numbers still fall within the normal range in most of the cases, as evidenced by our research. Herishanu et al. assessed the effect of leukocytosis in individuals exclusively with obesity (40).

Samocha-Bonet et al. (41) demonstrated that females with obesity had significantly elevated platelet counts compared to normal-weight females, which may be attributed to higher body fat mass. Additionally, they did not observe a significant elevation in platelet counts in the male subgroups. Thus, based on their results, obesity may be associated with elevated platelet counts specifically in females with chronic inflammation (41).

The significantly elevated hematocrit levels in female patients with obesity as opposed to controls in our study is also consistent with the literature (42). Greater weight, stature, BMI, skinfold thickness, and lean body mass were found to be associated with higher hemoglobin and hematocrit levels (43). The use of adjusted reference intervals for the blood count constituents corresponding to different levels of overweight/obesity is already a matter of scientific interest (44).

According to our investigation, both serum amylase and lipase levels were significantly lower in patients compared to controls. Kondo et al. found that serum amylase and trypsin, but not lipase levels were significantly lower in subjects with obesity compared to those with ideal body weight. Furthermore, serum amylase had a significant inverse correlation with body weight. They assumed that lowered serum pancreatic enzymes in subjects with obesity are related to their diet intake (45). It has become evident that decreased levels of serum amylase are associated with more common conditions, such as obesity, diabetes, and metabolic syndrome (46). A systematic review showed that diabetes mellitus, excessive adiposity, and metabolic syndrome are characterized by low serum levels of amylase, lipase, and trypsin, so they can be considered as biomarkers of these metabolic disorders (47).

In the context of our inquiry, plasma 25OHD3/D2-vitamin levels were significantly lower in patients compared to controls. The collective data from meta-analyses (48–50), cross-sectional (51) and cohort (52) studies also consistently indicate a reverse relationship between vitamin D levels and body weight. The mechanisms underlying the association of low vitamin D in obesity involve factors such as volumetric dilution, sequestration into adipose tissue, reduced sunlight exposure, decreased vitamin D synthesis in adipose tissue and the liver (48), low physical activity, and intake of foods rich in vitamin D (53). The bone mineral content did not demonstrate any correlation with 25OHD3/D2-vitamin levels in the present study. It should be noted that there is a correlation between vitamin D supply and painful neuropathy (54, 55).

According to our investigation, phosphate levels were significantly lower in patients compared to controls. Serum phosphate level was also found to be inversely associated with BMI in women (56, 57). The inverse correlation between phosphorus levels and weight gain is influenced by the regulation of food consumption, thermogenesis, physical activity capacity, and energy usage (58). Food intake stimulates the release of insulin and necessitates the phosphorylation of proteins and carbohydrates leading to increased phosphate absorption from the extracellular blood serum into the liver and skeletal muscle, subsequently reducing serum phosphate levels (59).

In this study, the serum uric acid level was significantly higher in patients than in controls, which finding is also coherent with the literature (60, 61). Elevated levels of uric acid are known as a good predictor of underlying co-morbidities including diabetes, obesity, and hypertension (62, 63). It has also been reported to be associated with nonalcoholic steatohepatitis (64). Obesity is associated with impairment of liver function by a variety of mechanisms (65). In our study, the serum levels of the liver function marker ASAT/GOT were not significantly elevated in female patients with obesity compered to controls, while ALAT/GPT, GGT, and alkaline phosphatase (ALP) were all found to be significantly elevated. Similarly, a positive correlation was reported for abdominal obesity, ALAT/GPT and GGT in previous studies (66, 67).

Lipid abnormalities including elevated triglyceride, very-low-density lipoprotein, apolipoprotein B, LDL-cholesterol levels, low HDL-cholesterol, and apolipoprotein A-I levels are commonly observed in patients with obesity (68–71). In our study, we found a significant elevation in the serum levels of triglycerides and a significantly lower level of HDL-cholesterol in patients compared to controls.

Obesity is a recognized risk factor for developing carbohydrate metabolism disorders (72). Consistently, in our patient cohort, the prevalence of IGT as well as fasting serum blood glucose were significantly higher compared to the control group. Although HbA1c% is almost identical between the two groups, fasting insulin levels and insulin resistance characterizing HOMA-IR, tend to increase in the patient group without reaching the level of significance. We also found significantly elevated fasting plasma glucose in patients with obesity, a finding reinforcing the role of obesity as a contributing factor to carbohydrate metabolism disorders.

We found significantly elevated serum potassium levels in obese female patients compared to controls. It’s noteworthy that existing literature tends to present findings that are more contrary to this result. A cross-sectional study of 10 341 participants found that low serum potassium levels were significantly associated with the prevalence of metabolic syndrome in middle-aged and elderly Chinese individuals (73). Our results may be influenced by the fact that a significant proportion of patients had hypertension (23 vs. 0; p<0.001), and 13% of the patients were undergoing ACE inhibitor or ARB treatment.

## Conclusion

Our study revealed impairments in peripheral sensory neuronal and sudomotor function in female patients with obesity compared to controls with a normal BMI value. Additionally, parasympathetic cardiovascular autonomic dysfunction was indicated by the Valsalva-ratio. The correlation between BMI and 25OHD3/D2-vitamin levels underlines the need for vitamin D supplementation in this population.

To the best of our knowledge, our study initially indicates small fiber dysfunction in women suffering from obesity, without diabetes, using Neuropad® test methodology. Moreover, testing with the Neurometer® technique has provided the first confirmation of elevated thresholds in the median nerve, in female patients with obesity compared to controls across all tested frequencies. This suggests early impairment of large myelinated, small myelinated, and small unmyelinated sensory fibers.

The relationship between obesity and neuropathic lesions is complex and multifactorial. The prevalence and severity of neuropathic dysfunction in women with obesity can vary depending on factors such as age, comorbidities, genetic predisposition, and lifestyle factors. Further research should be conducted to provide more specific and detailed information on this topic.

## Limitations

Considering the results of patients and controls, it is imperative to take into account that significantly more patients took metformin and β-blockers compared to controls. Beta blockade decreases sympathetic nerve activity, so it might attenuate the degree of abnormality in the achieved reflex test results in the patient group. In the case of metformin, we can also expect a similar masking effect through optimizing carbohydrate metabolism.

A greater sample size may be needed to increase the statistical power of the study and allow for a more comprehensive investigation of the relationship between the presence of obesity and other potential factors and the presence and the potential changes in the neuropathic status. Further research is warranted to investigate the effect of lifestyle interventions including nutrition coaching and physical training on neuropathy.

## Data availability statement

The raw data supporting the conclusions of this article will be made available by the authors, without undue reservation.

## Ethics statement

The studies involving humans were approved by Hungarian Medical Research Council (approval No. 219 31891-5/2019/EÜIG). The studies were conducted in accordance with the local legislation and institutional requirements. The participants provided their written informed consent to participate in this study.

## Author contributions

NK: Writing – original draft, Writing – review & editing. JZ: Data curation, Methodology, Resources, Writing – original draft, Writing – review & editing. BL: Data curation, Project administration, Resources, Writing – review & editing. DS: Writing – review & editing. VM: Writing – review & editing. KW: Writing – review & editing. SL: Writing – review & editing. MS: Data curation, Formal analysis, Writing – review & editing. AM: Writing – review & editing. PK: Writing – review & editing. IB: Writing – original draft, Writing – review & editing. TV: Writing – review & editing. CL: Writing – original draft, Writing – review & editing. AV: Conceptualization, Data curation, Formal analysis, Funding acquisition, Investigation, Methodology, Project administration, Resources, Software, Supervision, Validation, Visualization, Writing – original draft, Writing – review & editing.
